# Knowledge of and Perceptions about Sexually Transmitted Diseases and Pregnancy: A Qualitative Study among Adolescent Students in Uganda

**Published:** 2007-09

**Authors:** Sunita Chacko, Walter Kipp, Lory Laing, Geoffrey Kabagambe

**Affiliations:** 1 Department of Public Health Sciences, Faculty of Medicine and Dentistry, University of Alberta, Edmonton, Alberta, Canada; 2 Institute of Public Health, Makerere University, Kampala, Uganda

**Keywords:** Adolescents, AIDS, Dual protection, HIV infection, Knowledge, attitudes, practices, Perceptions, Qualitative studies, Reproductive health, Sexually transmitted diseases, Uganda

## Abstract

This article reports the findings of a qualitative research study carried out in Kabarole district, western Uganda. Knowledge of and perceptions about HIV/AIDS and pregnancy and how both relate to one another were elucidated from eight focus-group discussions with 38 female and 32 male secondary students from four different schools. Widespread misinformation and misconceptions about contraceptives still exist as previously found in this area. There was a serious gap in knowledge and understanding of ‘dual protection’ against sexually transmitted diseases, including HIV/AIDS, and against pregnancy. Fertility was very highly valued, and many girls stated that they would want a child even if they were HIV-positive. Responses of girls showed that they were quite assertive in making decisions to use contraceptives. The reasons for students not being able to understand the interconnectedness of sexually transmitted diseases and pregnancy may lie in the fragmented fashion in which relevant health education is delivered through two separate programmes.

## INTRODUCTION

For adolescents in Uganda, infection with human immunodeficiency virus (HIV) and complications due to teenage pregnancy are two of the most perilous health hazards they can ever encounter. The Ugandan teenage pregnancy rate is one of the highest in sub-Saharan Africa: it has recently been reported that over one-third of females aged 15–17 years have had sexual intercourse, and 35% of girls aged 15–19 years were either pregnant or had already delivered a child (1). Sexually-active adolescents run a high risk of acquiring sexually transmitted infections (STIs), including HIV, through unprotected sexual intercourse. Despite a recent decline in the HIV prevalence in Uganda, 500,000 persons are still living with HIV/AIDS, and 50% of them are aged less than 25 years (2). These figures suggest that the risks of teenagers becoming pregnant and acquiring HIV infection are very real in Uganda.

Many African teenagers who have an unwanted pregnancy consider abortion as an option. For example, it was found that, in Dar Es Salaam, Tanzania, pregnant teenagers used abortion as a contraceptive method because cultural and practical barriers accessing contraception presented greater obstacles (shame) than the risk (fear) of having an abortion (3). Illegal abortions in many countries like Uganda are connected with high morbidity, as most are performed in an unsafe manner by unskilled individuals (3). Unsafe abortion also threatens the safeguard of fertility because it is considered an important risk factor for becoming infertile (4). In South Africa, pregnant teenagers, who decided to continue with a pregnancy, attended antenatal clinics often only in the late stages of their pregnancy which left them exposed to other health risks, such as hypertension and eclampsia (5). Pregnant teenagers also have a higher risk of birth-related complications due to their young age which may result in additional threats to their fertility.

One of the most important ways to protect adolescents from acquiring an STI or HIV and becoming pregnant is dual protection. Dual protection has been advocated by many professionals familiar with both family-planning and HIV/AIDS-control services. Dual protection can be achieved through: (a) using a male or female condom together with another contraceptive method, e.g. oral contraceptives, (b) using a non-barrier method (e.g. oral contraceptives) and mutual monogamy between HIV-negative partners, and (c) non-penetrative sex (4).

Knowledge about dual protection among users is generally limited. Searching by the key word ‘dual protection’ resulted in only 12 articles in the Medline/PubMed database from 1966 to present. We found only three studies on this topic from developing countries, all of them from South Africa. Two were original research studies (6,7), while the third one was based solely on secondary analysis of data from the South African Demographic and Health Survey (8). All of these studies targeted the general population. None of these studies were tailored towards the specific reproductive health needs of adolescents. The three studies concluded that knowledge and use of dual protection against STI/HIV and pregnancy was low and was rarely promoted by reproductive healthcare workers.

We conducted the present study to obtain more information on knowledge of and perceptions about dual contraception in Ugandan adolescents. We used a qualitative methodology for data collection. Qualitative approaches have the advantage of providing a means to acknowledge the concerns of a target population and of developing a partnership based on trust. We worked closely with officials of the Health Department of Kabarole district and involved one research assistant from an adjacent community to strengthen this partnership. The study was conducted from September 2003 to December 2003.

### Background information

Kabarole district has an estimated population of 397,422, and the literacy rate is 49% (9). The population has a low socioeconomic status with subsistence agriculture as the main source of income. The district has one of the highest total fertility rates (TFRs) (8.0) in the country. Similarly, the prevalence (around 12%) of HIV characterizes Kabarole as a high-prevalence district for HIV. The high level of occurrence for both HIV infection and pregnancy, especially in young people, highlights Kabarole as a district where the high risk for a pregnancy in HIV/AIDS-affected young people is above the national average. This poses a serious public-health problem which needs more urgent attention and which is being addressed through the reproductive health programme for adolescents.

The District Health Management Team (DHMT) delivers reproductive health services in Kabarole district within the national framework. Adolescent-friendly services are one of the objectives in the Kabarole District Health Plan. Family-planning and HIV/AIDS-control services are delivered through separate programmes at the district and health unit level with different programme staff and different clinic hours. Oral contraceptives, Depo-Provera, intrauterine devices (IUDs), and condoms are available for adolescents. Vasectomy and tubal ligation services are offered in hospitals for adults who have completed their family size. Usually, the supply of contraceptives is reliable, but occasional shortages do occur.

Despite the insufficient integration of HIV/AIDS and family planning into one programme, the DHMT has made progress in the past years in providing young people with contraceptives, including condoms, by making them more widely available and accessible. One way of providing condoms more discreetly was the introduction of condom machines which operate by inserting a coin and which are placed in convenient and inconspicuous locations. These efforts have resulted in an increased use of contraceptives by adolescents: (a) the use of condoms increased from 48% in 1996 to 73% in 2001 (10); (b) the use of hormonal contraceptives increased from 0.5% in 1999 to 10% in 2005 as shown in district health reports; and (c) the HIV prevalence declined from 33% in 1991 to 9% in 1997 and from 9% in 1997 to 6% in 2004 in the age-group of 15–19 years (11,12).

Sex education for secondary school students is provided through the School Health Education Programme (SHEP). The District Health Educator and his assistants regularly visit schools. In addition, the “Presidential Initiative on Communicating to Young People about HIV/AIDS” (PIASCY), developed in 2002/2003, was introduced in 2005 (13). The PIASCY has a school-arm, but also focuses on out-of-school youths which have been neglected in the past. Teachers teach the teaching modules in schools, which are also delivered through regional two-day gatherings of 1,000 adolescents or more. Other sources of reproductive health information are peers, radio messages broadcast by the local radio station with special messages for young people, and newspapers. ‘Straight Talk’ is a newspaper insert, which is very popular and a trusted source of information among adolescents. It is produced by a registered non-governmental organization (NGO) and has tackled controversial issues openly (for example non-invasive sex). Previous studies in Kabarole district have shown that communication between parents and their adolescent children about reproductive issues is inadequate (14,15). In the recent past, some groups claim that the Ugandan Government has changed its approach to HIV/AIDS prevention in stressing abstinence as the primary HIV/AIDS-prevention strategy which they claim is underwritten primarily by the United States Government (16). On the other hand, the Ugandan Ministry of Health ordered 50 million condoms in 2005, which seems to indicate that promotion of condom is considered equally as important.

### Research questions

To explore adolescent views on HIV and pregnancy, the following research questions were investigated: (a) How do Ugandan adolescents view pregnancy and its prevention?; (b) How do Ugandan adolescents view HIV infection and its prevention?; and (c) Do Ugandan adolescents make a connection between the issues of pregnancy and HIV infection and what do they know about dual protection against sexually transmitted diseases/HIV infection and pregnancy?

## MATERIALS AND METHODS

An exploratory, qualitative study of male and female secondary students in selected schools in Fort Portal, Uganda, was undertaken. Data were collected through focus-group discussions (FGDs). The FGDs were held in available classrooms and out-of-hearing distance of non-participants and with no teachers present to maintain privacy. Each question was asked to the group in general, and then each participant was given the opportunity to respond or pass. At the end of each FGD, a question period was held, whereby students could ask any question they wanted about any related topic.

### Study sample

The study sample included adolescents aged 14–18 years attending secondary schools in Fort Portal, Kabarole district. All four secondary schools in Fort Portal were included. Sampling of students was based on a convenience sample. The research assistant informed the students about the study and asked them to report to the headmaster if they were willing to participate. In total, 38 female and 32 male adolescents agreed to participate in the study. Participants were divided into eight focus groups. Two focus groups were conducted in a boarding school for male students, two were held in a boarding school for female students, and four were held in two mixed day schools. Each focus group consisted of 7–10 students. All focus groups were gender-specific (male only and female only) to increase comfort levels of students involved.

### Interview questions

A focus-group questionnaire was created to serve as a basic discussion guide. Topics were arranged in a seemingly logical order, dealing with pregnancy, then HIV, then questions that dealt with both the issues, such as questions on dual protection, or mother to child transmission. Dual protection was defined as strategies that provide a simultaneous safeguard against both pregnancy and STIs (17). The interview guide and schedules were reviewed with Ugandan researchers and health officials. The FGDs were held in English, since English is the language of instruction in all schools in the district and the fact that all students speak English very well. The first author (who is of East Indian descent) served as a moderator for the FGDs, and a Ugandan research assistant, who was trained in qualitative assessments, served as a note-taker.

### Data recording and analysis

Each FGD lasted approximately two hours and was audio-taped and then transcribed. The first author kept a notebook of thoughts and ideas expressed by the participants and observations in the group that provided additional data. Upon completion of data collection, all data were compiled from audio tapes, recorded notes, and the primary researcher's observation notebook. In transcribing the data, an attempt was made to transcribe the discussions verbatim, outlining emphasized words, pauses, and other such vocal activities as recommended by Roth (18). After transcription, and an overall reading and surface analysis of the transcript was completed, the data were then organized by question and response sets and divided up as male and female responses to each question. Analysis of data followed the method outlined by Roth (18).

Categorization was done which consisted of looking at the statements for similar ideas. These categories were given titles that summarized the main idea they encompassed. Thirteen categories were created which had an average of 78% agreement in coding by a second reviewer. Throughout the entire process, an audit trail was developed through continuous collaboration between the researchers and the health officials. This process of creating an audit trail allowed for transparency throughout the data analysis.

### Ethical considerations

Ethical approval for the study was accorded by the Health Ethics Review Board Panel B at the University of Alberta. Upon arrival in Uganda, further ethical approval was obtained from the Uganda National Council for Science and Technology, Kampala. Permission was also obtained from the District Director of Health Services and the District Educational Officer in Fort Portal. The headmaster of each school gave verbal consent for the participation of students on behalf of their guardians. At the beginning of each FGD, written consent was obtained from each, allowing the students the opportunity to withdraw at any time from the study. Students were assured that, in the final report, all comments would remain anonymous and that their participation would not be divulged to others outside the discussion room.

## RESULTS

Analysis of the transcripts revealed 13 categories with 10 categories identified simultaneously by two independent reviewers of the transcripts. The 10 categories were as follows: Doubts about contraceptive efficacy; mistrust in relationships; general fear/anxiety/worries; embarrassment and shyness; role of parents; value of fertility; spirituality and religion; misconceptions in reproduction; gaps in knowledge; and female assertiveness. The five most important categories (according to frequency of reporting) are presented as follows:

### Doubts about contraceptive efficacy still persist

Most focus-group participants expressed skcepticism when discussing common contraceptives, such as condoms or birth-control pills. While in many cases, the students stated that they would still use some form of contraceptive (usually condoms), many felt that using contraception was not highly effective and that they were still putting themselves in danger. A couple of students also expressed the opinion that condoms were to be used by those who were promiscuous rather than ‘proper’ individuals, assigning a certain moral designation to those who might choose to use them:

“I could just use it (condom) for emergency because it only protects one for getting protection against pregnancy, but it does not protect against AIDS or other diseases and so I would just use it in emergency. I would use to not get pregnant, but I would know I am likely to get AIDS or HIV using it” (male student).

“For me, I think a condom was meant for those people, used by mobile women or men [referring to transient or promiscuous individuals] because for those one who go in discos, when you reach in there, there is a girl who is drunk, he tries to do what rape ….” (female student).

Many students when asked specifically about condoms had comprehensive explanations as to why they believed that condoms were not effective. This ranged from conducting their own experiments on condoms (i.e. pouring water into them to see if it passed through) to citing studies that they had heard about, or seen on television about the effectiveness of condoms. Many students used the phrase ‘condoms are not 100% effective’ which is, in fact, true; however, their explanation as to why condoms were not 100% effective was in most cases lacking factual information.

### Trust/mistrust in relationship determines choice for contraceptives

This idea that emerged in discussion was that contraceptive methods used depended on the trust level in the relationship. When asked specifically if the contraceptive method depended on the type of relationship, many students felt that as the relationship grew to be long term, it was more appropriate to change methods to the birth-control pill, as condoms symbolized limited trust of your partner and were more appropriate for newer relationships where trust was yet to be established.

“ … I would prefer using pills because at least people have told me that at least if you use a condom you will not feel good sweetness. I have not experienced this, but I would prefer using a pill, because if you were to have your girlfriend and you really use her, you will need to get the best of the best enjoy the best of the best (laughter) so I would prefer using pills, the relationship stays if we are trusting each other, I don't think she has HIV or myself, we are trusting each other and become one (laughter from group). So, she uses pills, and we meet in that case, we are avoiding pregnancy” (male student).

In regard to the suggestion by a female student to use condoms, males saw this as a female attempt to protect the male from HIV infection or as an indication that the girls suspected them of being HIV-positive. While a number of boys saw it as a sign of responsibility on the part of the girl, many viewed the idea of females suggesting condoms with wariness:

“Ok, definitely, if a girl tells me to use condoms, I must respond because also I have a hope that she may be having HIV and she does not want to spread that to me, and therefore if she tells me to use a condom, I have to protect myself, therefore I can respond ….” (male student).

### Value of fertility is high

When asked if they would still want to have a child if they are HIV-positive, many girls responded that they would, in fact, have a child and then referred to the value of leaving behind a legacy, or someone to be remembered by:

“I may want to produce a child even if I am infected because when I die, I leave that to my fellow relatives, they may keep remembering me, when they see him or her, this one is the daughter of (name) and they keep on remembering me” (female student).

This attitude was also echoed by young men. While some students did reflect on the fact that the baby would have a difficult life and could be HIV-infected and show concern for who would take care of the baby, others felt strongly that, in the event that they were to be HIV-infected in their lifetime, their parents could look after the child after they were gone, and as long as there was someone to take care of the baby, they would go ahead with the pregnancy. Many youths felt that going to the hospital and receiving drug therapy so that the baby would not be HIV-infected was a readily available and simple solution as shown in the comment below:

“For me I can wish to have a child because they say that a girl who is having a child can produce a baby who is HIV-free, when they produce a baby through proper doctors, because there is this programme—Prevention of Mother to Child Transmission of HIV. The mother can produce a child very simply without getting HIV. So when I leave behind my child, I would not have died completely, I would have left a person in the world” (female student).

### Reproductive knowledge is lacking and misinformation persists

Adolescents in all focus groups held a wide array of misinformation. While some misinformation might be considered relatively harmless, it is important to realize that misinformation in actuality forms the individual's concept of reality. Some of the most common misinformation held by students dealt with contraceptives, especially condoms and birth-control pills, as illustrated by the following statements:

“I have heard many people prophecy that condoms are good what, especially the government on radios you hear them advertising that condoms are good and that they stop pregnancy and the like … I made a good observation. I found that I shouldn't use a condom. I shouldn't because in the research that I made, I asked several people, they said when you use a condom for example we boys, your penis may not be all that big enough to accommodate the size … it may not fit you, may not be big enough. One of them told me if you are not careful, it may skip and go into the vagina of the girl with the sperm. I find that situation can be confusing … so I still doubt if I use it, I may get AIDS and all those things make me conclude that the condom isn't good” (male student).

“On my side using condoms with my partner for a long time is bad, because you can get diseases from using condoms like cancer, but my partner can get blood check-ups [i.e. HIV test] … then can go for injections [injectable birth control] (male student).

One of the main questions of the study explored during the FGDs was relating to the concept of dual protection. One of the problems encountered with this question was the fact that students did not understand why protection against pregnancy and HIV infection needs two different methods. However, after this concept of using two methods at the same time was explained in detail, most students did not understand its relevance. Some students felt that it was redundant and unnecessary to use two forms of protection. This opinion is summarized by one of the only statements recorded:

“If you are using pills to prevent pregnancy, at the same time using condom to prevent both, so why apply two?” (female student).

In regard to non-invasive sex, girls did not ask any questions. A few males commented on masturbation with the following statements in which they believed that the release of sperm from the body could potentially decrease the viral count that existed within the body:

“Don't you think that masturbation can reduce the number of viruses in the body?” (male student).

“This Straight Talk Club say that when you feel emotions, you revert to masturbation … what is that? Is it good, can it bring some HIV? But I think that it is impossible because when you are with a partner, you can't apply that” (male student).

Comments on non-invasive sex were received from boys only. It was surprising how relatively the noninhibited boys were talking about it. It is also important to note that this topic was brought up by the participants themselves and not by the moderator of the FGDs. This shows that there is obviously a need for information on this topic, which is rarely tackled and which is not part of the official Uganda school curriculum on the prevention of HIV/AIDS.

### Female assertiveness is beginning to emerge around contraceptive-use

One of the interesting behaviours witnessed among female participants was that many young girls felt comfortable asserting themselves in regard to contraceptive-use and discussing their concerns about pregnancy with their boyfriend. Most comments that demonstrated assertiveness were in response to questions regarding comfort level in discussing protection and adverse consequences of unprotected sex:

“For me I understand, most boys get advantage of shy girls, and playing sex. For me if I was the one and I had a boy lover or boyfriend, I would call him aside and we discuss about HIV, unwanted pregnancy and after discussing about it in case he asks for sex at my age … I just advise him and tell him that it is still early and time is still there and we have to wait until we finish studies and that's when we can go into those things.

Upon further questioning, the interviewer asked: [You would not worry about telling him that, that maybe he would go find another girl?] to which the adolescent responded:

“Let him go, there are many (laughs)” (female student).

“If you have a boyfriend, maybe if I had one, I would just be free to him without fearing him or being shy. Because the moment you fear him that is when you fall into problems. You share ideas and tell him what is wrong and what is right. I think I would not fear him and maybe I would advise any other teenagers who are around, to put in their minds that thing of not fearing each other” (female student).

“For my case, I've got one (i.e. a boyfriend), and I don't feel shy when I'm talking to him about the dangers of getting pregnant so I advise him if we are to have sex, we use a condom” (female student).

Some female students commented that they would use their own protection if the boy disagreed. Many girls displayed a lack of concern when asked about the chance of a boy ‘dropping’ them if she did not comply with his demands. This is a positive step, demonstrating that young women seem to have a sense of self-respect and self-worth and that they are not reliant on their partners to the point where it can be detrimental to their own health and well-being.

## DISCUSSION

The results of our study provide additional evidence of the urgent need to continue to educate adolescents about reproductive health issues. The findings of the study indicate that young people have great worries about their reproductive life and mistrust the available methods. Many students stated that, if they became pregnant or HIV-infected, they would have nobody to turn to help in this crisis situation, as parents are often not considered a source of information or a source of help. This, not a new finding, has been identified in several previous studies from Kabarole district and from other parts of Uganda (14,15,19,20). What is new from this study is the general lack of awareness of young people about how HIV/AIDS and pregnancy are interconnected and how safe sex and family-planning methods influence and/or complement one another.

When the participants were asked about the association between preventing HIV/AIDS and preventing pregnancy, there was silence and only one comment was received. This indicates to us that the connectivity of these issues is not understood. The concept of ‘dual protection’, when explained in detail, was completely unknown to them. In addition, the finding that condom-use was viewed by most participants as a method of preventing HIV infection and not preventing pregnancy and that oral contraceptives were viewed as a method to prevent pregnancy only without any understanding on how oral contraceptives relate to HIV infection, underlines this knowledge gap. Dual protection against unintended pregnancy and STIs, including HIV, is considered crucial, especially for adolescents, to have sexual relationships without suffering major health consequences (4). Dual protection can also be achieved through non-penetrative sex. The knowledge about non-invasive sex was very limited in our student population, but the need for more information on this topic was clearly expressed, and the boys showed that it is possible in Kabarole district to talk about it in front of others. Non-invasive sex has been widely neglected in the promotion of safe sex, although it remains a potentially important area for the ongoing improvements of adolescent reproductive health programmes, according to Brady (4).

The fact that the topic of non-invasive sex was started by the adolescents themselves and not by the moderator, reinforces what Brady stated (4). It also demonstrated the great information need of adolescents from Kabarole on this topic and their readiness to discuss it among their peers.

The lack of understanding and confusion about HIV and pregnancy among young people is a great concern and is viewed by many health professionals involved in HIV/AIDS control and family planning as a consequence of delivering HIV/AIDS services and family-planning/safe motherhood services in a segregated fashion. Our study did not investigate this relationship, but Richey found, during visits to family-planning clinics in Tanzania, that family-planning clinic staff (doctors and nurses) hardly talked about HIV/AIDS and did so only if requested by clients (21). Others argued that competing bureaucracies in family planning and HIV/AIDS prevented the integration of both the programmes under one umbrella and that to date, many ‘new’ reproductive health programmes are still being implemented through the same structures of the old policies (22). The fragmented delivery of HIV/AIDS-control and family-planning programmes, which is taking place at all levels of programme administration is, in our opinion, largely a result of design of a vertical organizational programme and lack of funding for collaborative programmes. For example, Berer complains that, in the UNAIDS campaign for 2004 ‘Women and HIV’, the reproductive and sexual health needs and rights of women are largely absent in the campaign topics (23). When agencies and their professionals are not working together on these issues to create mutually beneficial messages and are delivering their health-education messages separately to the population, who can be surprised that these separate messages are not understood by the recipients as interrelated and complementary?

Fertility is very highly valued among male and female youths in Kabarole district. The desire for children is so great that a number of students felt strongly about going ahead with a pregnancy, knowing that HIV could pose a high risk to the baby. The comments of adolescents indicating that they are willing to carry a pregnancy despite the real chance of adverse effects on themselves and on the baby show that they do not fully comprehend the consequences of having a child with HIV infection and that more information is needed. The high value of fertility is understandable, as the literature clearly shows that infertility has serious implications for young women's lives. An example from India showed that infertility was deeply feared by young women and was perceived as one of the worst problems a woman might face (24). A similar situation exists in sub-Saharan Africa where repercussions from infertility (usually women are the only ones to be blamed) have serious social, economic and health impacts for women, particularly in traditional settings where the identity and status of women are linked to childbearing (21-23). To use this proscribed value as an entry point for reproductive health education, these programmes should include a component to preserve fertility (4).

One encouraging finding was that female adolescents were more assertive on how they approached reproductive decision-making and on how they plan to exercise some control within their sexual relationships. When compared to previous studies done on reproductive health among adolescents where the girls presented themselves in a more submissive attitude, this presents a significant change in the study area. The greater assertiveness of female adolescents was also demonstrated through their increased use of hormonal contraceptive in the age-group of 15–19 years over the past years: contraceptive prevalence increased from 0.5% in 1999 to 10% in 2005. We did not get substantive comments from male students on this topic, despite serious probing. It could have been very interesting to document the perspectives of male students on these statements made by the female students. The empowerment of women in Uganda has been on the radar screen in the recent past, as we heard repeatedly during our study from district officials and community leaders.

The limitations of the study were that: (a) study participants were restricted to adolescents who attend school; therefore, the study results do not represent the viewpoints of all youths in the region (e.g. such as those who are not attending school) and (b) some participants did not contribute as much as others to the discussion; therefore, their voices may not have been heard and considered compared to those who were talking more.

The strength of the study was that it gave adolescents in Kabarole district an opportunity to talk about sensitive and controversial topics regarding their sexual relationships. Their concerns about pregnancy and HIV infection were noted and discussed with the reproductive programme manager and the health team. In addition, it was a follow-up study to similar studies done earlier in the same population group which allowed valid comparisons between both the studies.

To forward the agenda of delivering appropriate reproductive health services for adolescents, i.e. which provides meaningful, appropriate, and integrated information and services to them, we recommend the following action steps to the District Health Department in Kabarole:

a. Counselling about both prevention of STI/HIV and pregnancy should always contain essential complementary messages from both the programmes as a default. This would require some retraining of health staff involved in HIV/AIDS and family-planning services and sensitizing them towards the importance of dual protection. This is mainly a question of developing the collaborative training programmes to create complementary messages. Dual protection to avoid STI/HIV and pregnancy in adolescents should be continuously and vigorously promoted through available channels of health education, preferred by adolescents, e.g. peer-education programmes, radio programmes, and newspaper inserts, such as ‘Straight Talk’ (an insert in the national newspaper ‘New Vision’).

b. The preservation of fertility, as expressed by our participants as a core value, should be fully integrated into both family-planning and HIV/AIDS-control programmes and be used as an entry point to better identify with adolescents and to better respond to their felt needs. The prevention of STIs by condom-use should also be promoted more as a means of preserving fertility by avoiding STIs. In addition, counselling young women who are infertile should be included in family-planning clinic services, recognizing the somewhat limited options available in Uganda for infertile young girls.

c. The district authorities should explore options for improving the administrative integration of HIV/AIDS/STIs and family-planning services. The Ugandan healthcare-delivery services are decentralized to the districts; thus, the districts have great flexibility in organizing their district healthcare delivery in a way which best serves their population. Reproductive health services can be improved through decentralization as shown in the Ghana example (25).

The action steps outlined do not require substantial additional funding. However, they do require a commitment by all, i.e. the health workers, the management, the politicians, the parents, the public, and the international donor community, to a more integrated service-delivery model. The international donor community should review its funding procedures and find creative ways to facilitate and require the integration of HIV/AIDS control and family planning by asking for practical examples, trials, and pilot studies to demonstrate best practice models. Dealing with an adolescent HIV-positive daughter having an HIV-positive child is one of the worst nightmares for any family. Yet, this nightmare still repeats itself many times over in Uganda and worldwide in developing countries, challenging the ability of healthcare workers to assist. This largely reflects the inability of the health system in the past many years to correct itself and deliver more effective reproductive healthcare for adolescents. This passive approach to one of the largest public-health problems definitely compromises the future of developing countries, and the commitment to resolving this as a priority issue must be tangible and immediate.
